# Evaluation of effectiveness of the PlasmaJet surgical device in the treatment of advanced stage ovarian cancer (PlaComOv-study): study protocol of a randomized controlled trial in the Netherlands

**DOI:** 10.1186/s12885-019-5275-3

**Published:** 2019-01-14

**Authors:** G. M. Nieuwenhuyzen-de Boer, W. Hofhuis, N. Reesink-Peters, P. C. Ewing-Graham, I. G. Schoots, J. J. Beltman, J. M. J. Piek, A. Baalbergen, G. S. Kooi, A. van Haaften, H. van Huisseling, L. Haans, M. Dorman, H. J. van Beekhuizen

**Affiliations:** 1 Department of Gynaecologic Oncology, Erasmus MC Cancer Institute, P.O. Box 2040, 3000 CA Rotterdam, The Netherlands; 2 Department of Obstetrics and Gynaecology, Franciscus Gasthuis and Vlietland, Rotterdam, The Netherlands; 3 Department of Obstetrics and Gynaecology, Medisch Spectrum Twente, Enschede, The Netherlands; 4 Department of Pathology, Erasmus MC Cancer Institute, Rotterdam, The Netherlands; 5 Department of Radiology & Nuclear Medicine, Erasmus MC University Medical Center, Rotterdam, The Netherlands; 6 Department of Obstetrics and Gynaecology, Leids University Medical Centre, Leiden, The Netherlands; 7 Department of Obstetrics and Gynaecology, Catharina Cancer Institute, Eindhoven, The Netherlands; 8 Department of Obstetrics and Gynaecology, Reinier de Graaf Gasthuis, Delft, The Netherlands; 9 Department of Obstetrics and Gynaecology, Albert Schweitzer Hospital, Dordrecht, The Netherlands; 10 Department of Obstetrics and Gynaecology, Haga Hospital, The Hague, The Netherlands; 11 Department of Obstetrics and Gynaecology, Groene Hart Hospital, Gouda, The Netherlands; 12 Department of Obstetrics and Gynaecology, Haags Medical Centre, The Hague, The Netherlands; 13 Department of Obstetrics and Gynaecology, Bravis Hospital, Bergen op Zoom, The Netherlands; 14 Department of Gynaecologic Oncology, Erasmus MC Cancer Institute, Rotterdam, The Netherlands

**Keywords:** Ovarian cancer, Cytoreductive surgery, PlasmaJet device, Quality of life, Cost-effectiveness, Histology

## Abstract

**Background:**

The most important goal for survival benefit of advanced stage ovarian cancer is to surgically remove all visible tumour, because complete cytoreductive surgery (CCS) has been shown to be associated with prolonged survival.

In a remarkable number of women, CCS is very challenging. Especially in women with many small metastases on the peritoneum and intestinal surface, conventional CCS with electrosurgery is not able to be “complete” in removing safely all visible tumour.

In this randomized controlled trail (RCT) we investigate whether the use of the PlasmaJet Surgical Device increases the rate of CCS, and whether this indeed leads to a longer progression free and overall survival.

The main research question is: does the use of the PlasmaJet Surgical Device in surgery for advanced stage ovarian cancer result in an increased number of complete cytoreductive surgeries when compared with conventional surgical techniques. Secondary study objectives are: 30-day morbidity, duration of surgery, blood loss, length of hospitalisation, Quality of Life, disease-free survival, overall survival, percentage colostomy, cost-effectiveness.

**Methods:**

The study design is a multicentre single-blinded superiority RCT in two university and nine non-university hospitals in The Netherlands. Three hundred and thirty women undergoing cytoreductive surgery for advanced stage ovarian carcinoma (FIGO Stage IIIB-IV) will be randomized into two arms: use of the PlasmaJet (intervention group) versus the use of standard surgical instruments combined with electrocoagulation (control group). The primary outcome is the rate of complete cytoreductive surgery in both groups.

Secondary study objectives are: 30-day morbidity, duration of surgery, blood loss, length of hospitalisation, Quality of Life, disease-free survival, overall survival, percentage colostomy, cost-effectiveness. Quality of life will be evaluated using validated questionnaires at baseline, at 1 and 6 months after surgery and at 1, 2, 3 and 4 years after surgery.

**Discussion:**

We hypothesize the additional value of the use of the PlasmaJet in CCS for advanced stage epithelial ovarian cancer. More knowledge about efficacy, side effects, recurrence rates, cost effectiveness and pathology findings after using the PlasmaJet Device is advocated. This RCT may aid in this void.

**Trial registration:**

Dutch Trial Register NTR6624. Registered 18 August 2017.

Medical Ethical Committee approval number: NL62035.078.17 (Medical Ethical Committee Erasmus Medical Centre Rotterdam).

## Background

Ovarian cancer is the seventh most common cancer in women worldwide with 239.000 new cases diagnosed in 2012. In The Netherlands 1325 patients were affected by ovarian cancer in 2016; of these 80% were diagnosed with advanced stage disease, for which surgical cytoreduction combined with chemotherapy is indicated [1–3]. During the last decade, surgical and chemotherapeutic treatment has not led to significant improvement in survival. In surgical treatment it is important that all visible tumour is removed (complete cytoreductive surgery, CCS) because the progression-free survival (PFS) and overall survival (OS) after complete cytoreduction is significantly longer than after optimal cytoreductive surgery, where tumour volume of up to 1cm^2^ remains in the abdomen [4–10]. In some cases it is impossible to achieve complete cytoreduction with conventional surgery due to the presence of many small tumour foci scattered on the intestines. Electrosurgery is unsuitable for tissues such as the intestine because of lateral thermal spread and depth of tissue destruction [11].

The PlasmaJet Surgery Device is an advanced energy system delivering pure plasma to the tissues. Plasma is a highly energized phase of gas which is short-lived and quickly dissipates at the targeted site of application, allowing controlled use [12, 13].

PlasmaJet is able to vaporize small tumour spots on intestine, mesentery, peritoneal surface, liver and spleen and is able to dissect peritoneum from the underlying tissue without muscle impulses and with less tissue damage than with conventional electrosurgery [11]. In the case series published on this subject application of the PlasmaJet during cytoreduction resulted in higher rates of CCS (79%) and fewer colostomies without any additional complications [14–20].

In this study, we will compare the success rate of CCS with the use of conventional surgery including electrocoagulation (control) with the addition of PlasmaJet Device (intervention) in a single blinded multicentre randomized controlled trial (RCT) to evaluate the effectiveness of the PlasmaJet when applied in the surgical treatment of women with advanced-stage ovarian cancer [21].

## Methods/design

### Setting and study population

This study is called the PlaComOv-study. It is an acronym for ‘Will the use of the PLAsmajet device improve the rate of COMplete cytoreductive surgery for advanced stage OVarian cancer.

In this study, 330 patients with a FIGO IIIB-IV epithelial ovarian cancer, carcinoma of the fallopian tube or extra-ovarian epithelial ovarian cancer(peritoneal cancer) in whom the surgical goal is to achieve complete cytoreduction will be included. Patients should to be fit for CCS and chemotherapy.

Patients from the following Dutch hospitals may be included: Albert Schweitzer (Dordrecht), Bravis (Bergen op Zoom), Catharina Cancer Institute (Eindhoven), Erasmus MC (Rotterdam), Franciscus Gasthuis and Vlietland (Rotterdam), Groene Hart Hospital (Gouda), Haags Medisch Centrum (Den Haag), Haga Hospital (Den Haag), Leids University MC (Leiden), Medisch Spectrum Twente (Enschede), Reinier de Graaf Groep (Delft).

All surgeons are trained and certified in the use of PlasmaJet during the preparation of the study.

This study will compare the complete cytoreductive surgery rate when using electrocoagulation only (standard) with that achieved with additional use of the PlasmaJet Surgical Device (intervention). We expect that use of the PlasmaJet during surgery will result in a higher rate of complete cytoreduction and fewer colostomies [14–20].

Standard therapy is primary cytoreductive (upfront) surgery followed by chemotherapy, or neoadjuvant chemotherapy followed by interval cytoreductive surgery. Standard chemotherapy comprises of 6 cycles of carboplatin and paclitaxel, with a duration of 21 days for each cycle [1]. In upfront cytoreductive surgery, all 6 cycles of chemotherapy are given after surgery. In interval cytoreductive surgery, 3 cycles of chemotherapy are administered prior to surgery and 3 cycles thereafter. Patients from both the upfront and interval cytoreductive groups may be included.

The standard of care is to reach complete cytoreduction in all women who are fit to undergo extensive surgery. This radical surgery may involve bowel surgery sometimes including colostomy. Electrocoagulation (Diathermy, LigaSure), scalpel and scissors are used during conventional surgery to remove visible tumour and to dissect tumour tissue from peritoneal surfaces. The disadvantage of electrocoagulation is the lateral thermal spread and the depth of tissue destruction, which render it unsuitable for use on the intestines. Electrocoagulation (Diathermy, LigaSure), scalpel, scissors and PlasmaJet are used when indicated during surgery in the intervention arm.

### Inclusion criteria

In order to be eligible to participate in this study, a subject must meet all of the following criteria:

- patients with epithelial ovarian, tuba or peritoneal carcinoma FIGO IIIB-IV who are fit enough to undergo radical cytoreductive surgery as discussed in the Tumorboard. Patients can either be scheduled for primary cytoreduction or for interval cytoreduction after neoadjuvant chemotherapy
- patients should understand the patient information form and sign informed consent
- pre-operative CT scan meets criteria for resectability

### Exclusion criteria

A potential subject who meets any of the following criteria will be excluded from participation in this study:

- patients who are not willing to participate or not able to give their informed consent (language barrier) and patients who are not willing to undergo extensive surgery
- patients who are unfit to undergo extensive surgery (assessed by gynaecologist and anaesthesiologist and discussed in Tumorboard)
- patients who are not fit enough to get the standard complete chemotherapy (six cycles carboplatin paclitaxel) (assessed by medical oncologist and discussed in Tumorboard)
- patients with a non-epithelial, borderline ovarian tumour or an ovarian metastasis of another primary tumour
- patients with recurrence of ovarian cancer.

#### Primary outcome

The primary study objective is to determine the rate of complete cytoreductive surgery in each group.

#### Secondary outcomes

- Complication rate (30 day-morbidity)
- Duration of surgery and hospital stay
- Blood loss during surgery and number of blood transfusions
- Number of partial bowel resections and colostomies
- Progression free survival [22]
- Overall survival [22]
- Quality of life (questionnaires filled in prior to surgery, at 1 and 6 months and at 1, 2, 3 and 4 years after surgery
- Accuracy of presurgical structured reporting of CT scans (according to a structured checklist). This will be compared with surgical findings (as recorded by a Gynaecological Oncologist immediately after surgery) and histological findings [23–28].
- Histology: depth of tissue destruction [29–32]
- Cost effectiveness analysis [33–37]: Costs per (complete) cytoreduction and costs per gained life year QALY
- Total number of chemotherapy courses during overall survival
- Comparison of completeness of surgery between both study groups according to an independent review of the operation field by photos

A histology review will be carried out in a subpopulation of 30 patients from the PlasmaJet group (15 primary cytoreductive surgery, 15 interval cytoreductive surgery). We will study at specific spots of macroscopic tumour during surgery. One spot will be vaporized with PlasmaJet Device and analysed at the presence of residual tumour. Another spot will be the control sample.

Our hypothesis is that vaporization by the PlasmaJet Device will result in less tissue damage than electrocoagulation and that we shall not find vital tumour cells in tissue treated with PlasmaJet.

#### Intervention group

In the intervention group, the PlasmaJet Device will be used if necessary as an additional device during cytoreductive surgery.

PlasmaJet Surgical Device uses neutral argon plasma to vaporize small tumour nodules with minimal collateral damage [11–14]. This device helps to achieve complete cytoreductive surgery in patients with advanced stage ovarian cancer, most particularly by ablating small tumour foci on the abdominal peritoneum, diaphragm, intestinal mesentery and bowel serosa.

#### Control group

In the control group, standard surgical instruments combined with electrocoagulation will be used during cytoreductive surgery.

### Assignment of intervention

The study will be explained verbally to the patient by the gynaecologist, and patients will receive written information in accordance with Good Clinical Practice guidelines. Those wishing to participate will sign an informed consent form and will be randomized preoperatively. It is not always possible to assess the presence and stage of ovarian cancer preoperative, and in some cases it is unknown whether enlarged ovaries are malignant. In these cases, women can be randomized preoperatively and the gynaecologist will decide during the surgery whether the patient is eligible to be included in the study, depending on the result of frozen section and tumour stage.

Computer randomization will be used to allocate patients to the intervention or control group. Randomization will be carried out in blocks of varying size prior to surgery. Inclusions will be stratified depending on high or low suspicion of peritoneal carcinomatosis (based on the pre-operative CT-scan), primary and interval cytoreductive surgery, and the use of OVHIPEC during surgery or not [38–40].

The RCT is single blinded: the patient does not know to which arm she has been assigned.

### Data collection

Coded data are stored both on paper and in an electronic database. Collected data are stored in a digital case report form (CRF). Raw data is available only to the principal and coordinating investigator.

Patient characteristics will be stored in ‘Open Clinica’ and analysed in SPSS.

**Fig. 1:**
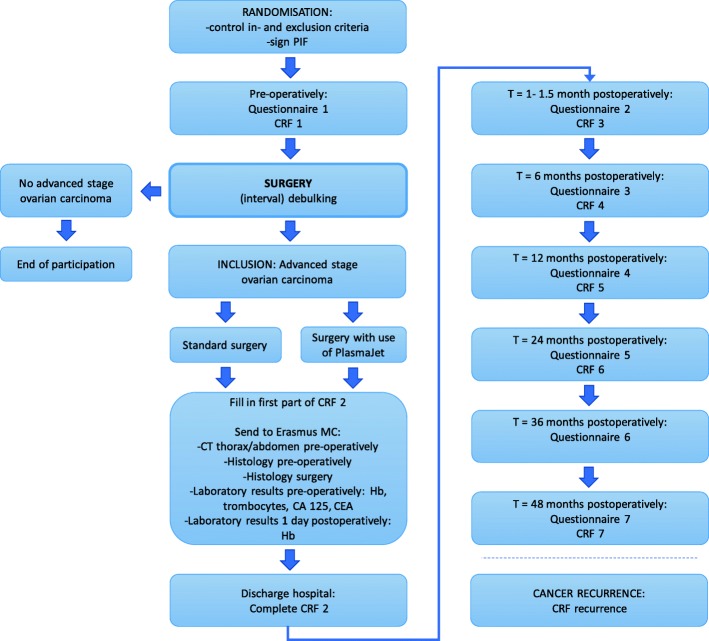
Flowchart of the study design

A CRF will be completed preoperatively, postoperatively-discharge and at 1½, 6, 12, 24 and 48 months postoperatively (Fig. 1).

Prior to surgery and at 1½, 6, 12, 24, 36 and 48 months postoperatively, a quality of life questionnaire will be completed (EORTC, QLQ-30 and EQ-5D) (Fig. 1).

The following data are recorded:

- *Preoperatively:*
  Patient characteristics, presence of germline mutations such as BRCA1 or 2, investigations carried out to make the diagnosis, outcome of structural reported CT-scan, chemotherapy, quality of life.
- *(Post)operatively:*
  Adverse effects of chemotherapy, operative parameters, tumour location, effectiveness of PlasmaJet during surgery, outcome of surgery, postoperative hospitalization, hospital discharge.
- *4–6 weeks follow-up:*
  Complications postoperatively, re-hospitalization, histology outcome, planned chemotherapy, quality of life.
- *6 months follow-up:*
  Complications of chemotherapy, re-hospitalization, indication of recurrence of malignancy, quality of life.
- *1,2,3 and 4 years follow-up:*
  Indication of recurrence of malignancy, new lines of chemotherapy administered, quality of life.

### Statistical considerations

Sample size calculations are based on our primary outcome measure. To demonstrate the additional value of the PlasmaJet, we assume an absolute increase of 15% in complete cytoreductive surgery in the PlasmaJet group (77% versus 62%). With a total type I error (alpha) of 5%, and a Type II error (beta) of 20%, 147 patients should be enrolled in each research arm.

Assuming a 12% loss of follow-up, a total of 330 patients should be recruited.

### Statistical analysis

The primary outcome measure, percentage complete surgery, will be calculated for each arm of the study together with a confidence interval based on the Wilson method. They will be compared using a chi-squared test with continuity correction. We will also calculate the risk difference. This will be presented with a 95% confidence interval (calculated using Newcombe’s method).

The study will be analysed according to the intention to treat principle. An exploratory subgroup analysis will be performed in a subset of patients with more than 50 lesions in the abdomen (peritoneal carcinomatosis), as complete cytoreductive surgery is not feasible for this group of patients. No multiplicity correction will be performed for these subgroup analyses.

Continuous secondary outcomes (duration of surgery, duration of hospital stay, blood loss) will be calculated using t-tests and the discrete variables (complication rate, bowel surgery, colostomies, number of chemotherapy courses) using chi-square tests using continuity correction.

All outcomes will be analysed using regression techniques. Progression-free and overall survival will be studied using the Kaplan-Meier method. Additionally Cox regression will be performed to study the influence of peritoneal carcinomatosis, complete, optimal or suboptimal cytoreduction. A *p*-value < 0.05 will be considered significant.

Multiple imputation using chained equations will be used for missing co-values.

The other study parameters will be analysed as follows:

1. Progression free survival (after 5 and 10 years) (Kaplan-Meier method)
2. Overall survival (after 5 and 10 years) (Kaplan-Meier method)
3. Cost per life year gained
4. Number of chemotherapy courses (chi-square tests using continuity correction)

No interim analysis for futility and effectiveness will be performed. A safety committee has been installed to monitor harm. The committee will receive data on safety and harm after each group of 50 consecutive patients and may advise stopping the trial for safety reasons after each analysis.

### Ethics and dissemination

The study has been approved by the Medical Ethical Committee of Erasmus Medical Centre Rotterdam. The study will be performed according to the standards outlined in the Declaration of Helsinki. Ethics committee approval has been granted.

Patients will receive verbal and written information from their gynaecologist during the intake for surgery. Randomization happens after signing of the Informed Consent.

Subjects can leave the study at any time for any reason if they wish to do so without any consequences. The investigator can decide to withdraw a subject from the study for urgent medical reasons. At this moment there are no specific criteria for withdrawal. After withdrawal the patient will be replaced since this is an intention to treat trial.

A monitoring plan is installed to ensure patients’ safety and the quality of this trial. Adverse events are recorded and reported by the sponsor through a local protocol. Study results will be offered for publication in international medical journals and on the website of the patient association for women with gynaecological cancer.

## Discussion

This study will contribute to the understanding of surgical treatment in patients with high stage ovarian cancer and will answer questions on implementation of the PlasmaJet Surgical Device. The results of this study will demonstrate whether the use of PlasmaJet Surgical Device will lead to a greater chance of complete cytoreductive surgery, and whether there is prolonged progression free and overall survival after operations conducted with this device.

The trial aims to study the efficacy of the PlasmaJet, side effects, survival rates and cost effectiveness, in comparison with conventional surgery. Pathology findings such as the presence of microscopic vital tumour after vaporisation with PlasmaJet and the depth of tissue damage after using the device will be studied.

A strength of this single blinded RCT is the use of questionnaires of Quality of Life (EORTC QLQ-30 and EQ-5D) and the involvement of the patient association of women with gynaecological cancer in The Netherlands.

### Trial status

Approved by Medical Ethical Committee Medical Ethical Committee Erasmus Medical Centre Rotterdam, The Netherlands on 20-11-2017. Recruitment started on 30-1-2018. Protocol version 3.0.

## Abbreviations

CCS: Complete Cytoreductive Surgery
CRF: Case Report Form
DMO: Data Management Office
NFU: Nederlandse Federatie van Universitair Medische Centra
OS: Overall Survival
PFS: Progression-Free Survival
RCT: Randomized Controlled Trial
SAE: Serious Adverse Events
WOG: Working party Oncological Gynaecology
